# Educators, Not Influencers: Health Content Creator Perspectives on Research Recruitment Collaboration

**DOI:** 10.1111/hex.70836

**Published:** 2026-08-20

**Authors:** Melissa L. Kramer, Bianca Serio, Aileen K. Ho, Katherine A. Finlay

**Affiliations:** ^1^ School of Psychology and Clinical Language Sciences University of Reading Reading Berkshire UK; ^2^ Live UTI Free Ltd. Dublin Co. Dublin Ireland

**Keywords:** content creators, digital health, health influencers, online health communities, research participation, research recruitment, women's health

## Abstract

**Context:**

Medical mistrust and delayed diagnoses drive patients to seek information outside of formal healthcare channels, meaning that traditional research recruitment methods then struggle to reach them. Social media health content creators with large followings possess an established online community presence and trust that could address this recruitment gap.

**Objective:**

To investigate how influential women's health content creators define their role, make decisions about content sharing, and evaluate research recruitment collaboration opportunities. This study aimed to understand how researchers can optimise study recruitment via influencer mechanisms.

**Methods:**

Semi‐structured interviews were conducted with 22 women's health influencers across 11 countries, in English, French, German and Italian. Influencer follower counts ranged from 1300 to over 700,000. Data were analysed using reflexive thematic analysis.

**Results:**

Six themes emerged: (1) Educators, not influencers; (2) mission as motivation; (3) leveraging reach; (4) care‐centred gatekeeping; (5) values‐led reciprocity; and (6) collaboration beyond logistics. Themes were examined against established influencer theories of Source Credibility, Similarity‐Attraction, and Social Exchange, which operated at both influencer‐follower and researcher‐influencer relationship levels.

**Discussion and Conclusions:**

Health content creators function as mission‐driven educators and careful gatekeepers who evaluate research opportunities against very specific criteria aligned with their own values. Their credibility, expertise, and shared experiences with followers have the potential to enable health behaviour change among their audience. They desire genuine research partnerships with early engagement and recognition of expertise. Strategic partnership models should therefore position creators as valuable collaborators in study design and recruitment rather than as advertising channels alone.

**Patient or Public Contribution:**

This study was conducted in collaboration with Live UTI Free Ltd., a patient‐led research and advocacy organisation. All the researchers have lived experience of recurrent or chronic UTI and other conditions within the focus of the study. Patient researchers co‐led all study stages: design, interview guide development, data collection, and analysis. Their lived experiences of seeking health information online about underserved conditions was essential for understanding how health content creators connect with online patient communities. Patient researchers conducted multilingual interviews and provided critical interpretation of findings. Members of the broader patient community identified health content creators as suitable interview participants.

## 1 Introduction

Clinical research recruitment faces persistent challenges that compromise study success, with ineffective recruitment frequently cited as a major reason for discontinuation of randomised controlled clinical trials [1]. Traditional recruitment methods that rely on physical means of communication, including in‐clinic and poster recruitment, have seen reduced reach and effectiveness with the shift towards telehealth and other digital health platforms [2, 3]. Beyond dedicated online health service delivery platforms, a more general rise in online health information seeking behaviour has occurred, particularly in areas of women's health and chronic illness where diagnosis is often delayed [4, 5, 6]. Protracted timeframes from new research discovery to implementation of findings in clinical practice have added to the medical mistrust observed among patient communities [7, 8]. This mistrust extends to concerns about the quality of online health‐related information, yet patients often seek information online prior to consulting a healthcare professional [9, 10]. These trends in online health information‐seeking behaviours and shifting patient communities further highlight why traditional research recruitment methods have failed. Researchers must develop new approaches to connect with online health information seekers in order to identify potential research participants.

Health information seekers are influenced by recommendations and endorsements within online patient communities, positioning influencers who are central to these communities as valuable mechanisms to connect with them [11]. These influential health content creators encompass diverse types: healthcare professionals who leverage clinical expertise, patient advocates who draw authority from lived experience with health conditions, and individuals who combine both professional credentials and personal health journeys [12]. They provide access to online communities where patient populations actively congregate around shared health experiences, shared interests, and the influencers themselves [13]. Social media platforms, particularly Instagram, TikTok, and YouTube, have become primary venues where health content creators share evidence‐based information, provide patient advocacy, and build communities around specific health conditions [12, 14]. Creators who utilise these platforms occupy a unique position to address research recruitment challenges: they provide access to online patient communities and health niches that traditional recruitment methods struggle to reach, and they hold credibility earned through authentic engagement with community members.

Credibility, access and authenticity must prompt action among online community members for research recruitment to succeed via these channels. Influential health content creators have been identified as effective conveyors of education and instigators of health behaviour change among followers, including promoting vaccination uptake, exercise intention and healthy dietary choices [13, 15, 16, 17]. Successful implementation of influencer‐based research recruitment has been evidenced in industry [12, 18], and more broadly, social media recruitment has shown enhanced reach and cost effectiveness compared to traditional methods [19]. However, most published studies focus on paid advertising campaigns [19] and academic investigation of influencer partnerships for research recruitment is in its infancy [20]. Deeper insights into whether online health influencers make effective recruitment partners will enable researchers to assess these channels as part of their research design.

### 1.1 Theoretical Mechanisms of Influencer Effectiveness

The effectiveness of social media influencers as recruitment partners can be explored through the lens of established theoretical frameworks that explain how influencers shape follower attitudes and behaviours. *Source Credibility Theory* posits that the persuasiveness of a message depends on the perceived expertise, trustworthiness, and authenticity of the source [21]. In the context of influential health content creators, credibility can stem from professional qualifications (e.g., physicians, nurses, health coaches), lived experience with health conditions, or both [22]. When followers perceive a content creator as credible, whether because of professional credentials, demonstrated expertise, authentic personal narratives, or community endorsement, they are more likely to trust the information shared, engage with recommended products or services, and adopt suggested behaviours [23, 24]. While Source Credibility Theory highlights why influencers are persuasive, it does not explain why followers choose to follow specific influencers in the first place.

*Similarity‐Attraction Theory* provides this missing piece, suggesting that individuals are more inclined to trust, form relationships with, and be influenced by others who share similar characteristics, attitudes, values, and experiences [22]. This mechanism, otherwise known as homophily, may be particularly powerful in health contexts, where shared lived experience of a condition creates perceived similarity between content creator and follower [25]. Unlike commercial influencers whose influence often stems from aspirational qualities or attractiveness, health content creators' influence may derive from being ‘like’ their followers; experiencing the same symptoms, navigating the same healthcare challenges, and acknowledging the same frustrations.

While Source Credibility Theory and Similarity‐Attraction Theory complement one another by explaining why followers trust the influencers they follow, and why they become followers, the reciprocal relationships observed between followers and influencers require further consideration. *Social Exchange Theory* fills this gap by proposing that influencer‐follower relationships are based on rational cost‐benefit analysis, with individuals seeking to maximise rewards and minimise costs in their interactions [26]. While various combinations of theoretical frameworks have been applied to commercial influencer marketing and health behaviour change, their relevance to research recruitment remains unexplored.

Moreover, existing literature focuses on influencer impact on followers, with little attention given to how influencers themselves make decisions about what to promote, with whom to partner, or how they perceive their communities. Understanding these aspects, and what influential health content creators perceive as valuable exchange is essential for developing sustainable collaboration models for research recruitment. This study explores how women's health and science content creators make decisions about what content to share and in which collaborations to participate, through the lens of the theories of Source Credibility, Similarity‐Attraction, and Social Exchange. Four research questions are addressed: (1) How do health content creators position themselves and establish credibility with their communities? (2) What motivates them to create content? (3) How do they make decisions about potential collaboration? (4) What are their perspectives on supporting research recruitment? Understanding these decision‐making processes is essential for strategically developing ethical, sustainable, and effective researcher‐creator partnerships that ultimately enhance health research participation.

## 2 Materials and Methods

### 2.1 Approach

This qualitative study employed online, semi‐structured interviews to allow for in‐depth exploration of health content creator experiences, motivations, and decision‐making processes. The study drew on three theoretical frameworks: Source Credibility Theory; Similarity‐Attraction Theory; and Social Exchange Theory. These complementary theories informed the interview guide development while permitting discovery of experiences and perspectives beyond these frameworks. The interview guide (see Supporting Information: S1: English interview topic guide) explored four key domains: (1) identity and role within the online community, (2) content creation motivations, (3) content decision‐making processes, (4) perspectives on research and research participation. Reporting adhered to Braun and Clarke's Reflexive Thematic Analysis Reporting Guidelines (RTARG) [27], and Big Q Qualitative Reporting Guidelines (BQQRG) [28].

Data analysis proceeded in two phases, commencing with inductive reflexive thematic analysis (RTA), with codes and themes generated directly from participant accounts. Second, the inductively‐derived themes were systematically examined against the three theories of Source Credibility, Similarity‐Attraction, and Social Exchange to identify alignments between participants' perspectives and established theoretical constructs explaining influencer effectiveness. This two‐phase process enabled findings to remain grounded in participants' experiences while also demonstrating how novel insights are positioned within existing theoretical frameworks of influencer theory.

### 2.2 Participants and Recruitment

A multilingual, multi‐platform approach was used to identify prospective health influencers for participation. Participants were eligible to take part in the study if they: (1) actively shared evidence‐based or advocacy content focused on women's health or science topics, (2) had a minimum of 1000 followers on at least one social media platform, and (3) were able to conduct an interview in English, Italian, German, or French. Country of residence and language were not applied as inclusion or stratification criteria, reflecting the borderless nature of social media audiences. Interview candidates were identified through three complementary strategies to capture creators with differing levels of visibility and embeddedness within online health communities. First, audience members in the Live UTI Free Ltd. (LUF) online community were asked to recommend online influencers in women's health and science. Second, the research team conducted systematic searches on TikTok and Instagram using keywords including: women's health, urinary tract infection, chronic UTI, recurrent UTI, menopause, pelvic health, bladder health, hormonal health, hormones, endometriosis, PCOS, urologist, gynaecologist, and obgyn. Keywords were translated into French, German and Italian to conduct language‐specific searches. Third, participants were invited to recommend other relevant health content creators, who were then approached by the research team.

In total, 209 candidates were identified, of whom 146 were contacted, 31 provided consent, 26 scheduled interviews, and 22 completed interviews (see Supporting Information: S2: Recruitment procedure flow diagram for numbers of candidates at each stage and in each language). Purposive selection among identified candidates prioritised a broad range of follower counts, a mix of credentialed and non‐credentialed creators, and coverage of diverse topics within women's health. While participants varied in professional background, the sample was homogeneous in its shared engagement in active health content creation within the women's health space, and all participants met an established follower‐count threshold defining influencer status [29]. Initial contact was made individually with each prospective participant in their native language. Contact was initiated either via the LUF Instagram or TikTok profile to demonstrate credibility within the online health community, or via the university email system to establish research credibility. Non‐responsive prospects were followed up once. No compensation, payment, or gifts were offered to content creators for study participation.

### 2.3 Procedures

A semi‐structured interview schedule was developed by the research team, in close collaboration with expert patient researchers from LUF (https://liveutifree.com), a patient research organisation focused on female pelvic health. The interview schedule was piloted in an interview in English with a women's health content creator who met the inclusion criteria and was known to the research team. Minor wording changes were then made to enhance clarity and the schedule was translated into Italian, French and German to enable participation in four languages, facilitating enhanced inclusivity.

All participants provided written informed consent prior to interviews. Identifying information was removed from transcripts and pseudonyms were assigned. Follower counts are reported as ranges to further protect identities; these reflect accepted definitions of influencer types: (1) Nano Influencers (1–10 K followers); (2) Micro Influencers (10–100 K followers); 3) Macro Influencers (100 K–1 M followers) [29]. Upon consent completion, participants received an interview scheduling link. Interviews were conducted via Zoom by the second author (BS), a multilingual health researcher fluent in English, Italian, German, and French. The interview language was selected by participants to ensure they could express themselves fully and comfortably. Duration of the interviews was between 35 and 83 min. All interviews were audio‐recorded with participant consent and transcribed using Otter.ai for English interviews and AudioTranscription.ai for the Italian, French and German interviews. Transcriptions were closely checked by the multilingual researcher who conducted the interviews. Interview participants were invited to review their transcripts for accuracy.

### 2.4 Data Analysis

RTA following Braun and Clarke's six‐phase approach was used [30]. Familiarisation with the data (1) was achieved through transcript checking following each interview, and via repeated reading of transcripts. Initial coding (2) was conducted by the lead researcher (MK) using spreadsheets, with codes generated both inductively from the data and deductively from the research questions and interview guide [31]. Themes were developed (3) through an iterative process of grouping related codes, identifying patterns, and refining theme boundaries through ongoing discussion and refinement. The full research team contributed by reviewing emerging codes and themes (4), naming and defining them (5), and integrating them into the final manuscript (6).

The sample of 22 participants was appropriate for the study's aims, providing rich, detailed accounts from health content creators across diverse contexts: 11 countries, four languages, follower counts ranging from 1300 to over 700,000, and varied professional backgrounds (medical professionals, allied health, patient advocates, scientists). This diversity enabled thorough exploration of decision‐making processes across different creator contexts and community types. Consistent with RTA, the concept of data saturation was not applied, as RTA prioritises interpretive depth and acknowledges that themes are actively constructed by researchers rather than emerging naturally from data at a saturation point [32].

### 2.5 Rigour and Reflexivity

Rigour was established through systematic and transparent analytical processes. The lead researcher (M.K.) maintained a reflexive log throughout data collection and analysis, documenting interpretive decisions, reactions to participant accounts, and how the researcher's positioning as an insider influenced engagement with the data. All coding decisions and theme development were discussed extensively with the research team, with disagreements resolved through collaborative interpretation rather than seeking consensus. Transcripts were returned to participants for accuracy checking only, consistent with RTA's epistemological stance that researcher interpretation is a valid form of knowledge production [28].

The lead researcher maintains a multifaceted position as an academic researcher, a health marketing specialist and the founder of a patient research and advocacy organisation. The research team comprised the lead researcher, two Chartered Psychologists and a multilingual researcher who conducted the interviews. The lead researcher's intersecting positions brought insider knowledge of online health communities and the practical challenges of research recruitment, shaping the analysis toward an attentiveness to creators' expertise and legitimacy as community figures throughout data collection and analysis. Consistent with a Big Q, constructivist approach, these varied positions were treated as interpretive resources that shaped and enriched engagement with participants' accounts.

## 3 Results

### 3.1 Participant Characteristics

Interviews were conducted between 12 June and 17 September 2025 with participants from 11 countries comprising Australia, Austria, Canada, France, Germany, Italy, Kenya, Switzerland, Tunisia, the UK, and the United States.

At the time of recruitment, all participants shared content primarily on Instagram and/or TikTok and had follower counts ranging from 1300 to over 700,000 on a single platform. The majority of participants held medical or allied health qualifications (15 participants, 68.18%), while others were expert patients, scientists, or held coaching qualifications or a degree in an unrelated field (7 participants, 31.82%). Participants had been sharing content for 6 months to 19 years, covering niche topics within women's health, including bladder health, gynaecology, hormones and pelvic health conditions. See Table 1 for detailed participant characteristics. Country of residence and language are reported for descriptive purposes only, consistent with the approach described in Section 2.3.

**Table 1:** Participant characteristics.

Participant pseudonym	Interview language	Country of residence	Highest follower platform & number range	Self described content focus	Confirmed qualifications
Lena	German	Austria	Instagram 1–10 K	Bladder health, with a focus on urinary tract infections	Expert patient
Ela	German	Germany	Instagram 1–10 K	Women's health with a focus on hormones and body‐oriented trauma support	Certified Naturopath
Cate	English	Australia	TikTok 100 K–1 M	Hashimoto's, gut health	Health coach, Nutritionist, Clinical Hypnotherapist, Coach, Mast of SIBO
Cassie	English	Canada	Instagram 10–100 K	Integrative approach to health: Gut health, hormone health, fertility, nervous system dysregulation	Licensed Naturopath
Theresa	English	Canada	Instagram 10–100 K	Pelvic health conditions	Patient Navigator Certification
Madison	English	Canada	TikTok 10–100 K	Pelvic health	Registered Physiotherapist
Alina	English	Kenya	TikTok 10–100 K	Reproductive health	Kenya registered community health nurse, CNLEX with Illinois, US.
Esther	English	Kenya	Instagram 10–100 K	General and preventative health	General Practitioner
Joanna	English	United Kingdom	Instagram 10–100 K	Mind‐body connection, health psychology and health experiences	Accredited Health Psychologist, Psychotherapist with the BA, BCP
Rebekah	English	United Kingdom	Instagram 100 K–1 M	Impact of hormones on health of women	General Practitioner/Physician, Scientist
Odette	English	United Kingdom	Instagram 1–10 K	Female hormones, energy efficiency and sport	Medical doctor, MRCP
June	English	United Kingdom	TikTok10–100 K	Menopause	CPD accredited menopause coach
Maggie	English	United Kingdom	Instagram 10–100 K	PMDD, PMS, endometriosis, PCOS, hormones, neurodivergence	Scientist, PhD researcher (female hormones)
Jade	English	United States	Instagram 100 K–1 M	Sexual function, hormones and continence	Board Certified Urologist, FPMRS
Louise	French	France	Instagram 1–10 K	Dyspareunia, vulvodynia, vaginismus, endometriosis ‐ disorders related to the intimate area	Former patient
Aurélie	French	France	TikTok 10–100 K	Menstrual education	Practitioner in Neurocognitive and Behavioural Approach
Justine	French	France	Instagram 1–10 K	Endometriosis	Expert patient advisor; Studying Psychology
Chloé	French	Switzerland	Instagram 10–100 K	Conditions related to painful intercourse (dyspareunia), including vaginismus, vulvodynia and others	Psychosexologist
Mila	French	Tunisia	Instagram 1–10 K	Menopause	Obstetrician‐gynaecologist, Master's in Reproductive Health, CEC in Senology
Beatrice	Italian	Italy	Instagram 10–100 K	Female urogenital health: vulvodynia, cystitis, Candida, chronic pelvic pain, sexual pain.	Degree in unrelated field
Regina	Italian	Italy	Instagram 10–100 K	Gynaecology and obstetrics	Specialist in Obstetrics and Gynaecology, Professor of Gynaecology
Aurora	Italian	Italy	Instagram 10–100 K	Under‐recognised conditions within female genital health	Registered Nurse, Degree in Nursing Sciences, Naturopath Certificates

*Note:* Health content creators from 11 countries participated in semi‐structured interviews conducted in English, German, French, or Italian. Follower numbers represent participant status at the time of recruitment and are presented as ranges to protect anonymity: Nano Influencers (1–10K followers), Micro Influencers (10–100K followers), Macro Influencers (100K–1M followers).

### 3.2 Overview of Themes

Our reflexive thematic analysis revealed six themes that characterise how participants perceive their role in, and interaction with, the online community: (1) Educators, not influencers; (2) mission as motivation; (3) leveraging reach; (4) care‐centred gatekeeping; (5) values‐led reciprocity; and (6) collaboration beyond logistics.

### 3.3 Theme 1: Educators, Not Influencers

Most participants rejected the ‘influencer’ label, instead positioning themselves as educators, advocates, disseminators, communicators, health professionals or a combination of these.

Health educator and health communicator. I don't like the term influencer, because I feel like it can be misconstrued. So health advocate, health communicator, health educator.

(Esther, Kenya)

I don't know if I'm like, trying to influence people, per se, as more like, I think of it more as educating and empowering people to learn more about their own health so that they can take, you know, action.

(Mila, Tunisia)

Many found the term ‘influencer’ inadequate, and some found it offensive, associating it with commercial manipulation, lack of credibility, and superficial content.

I don't use it because for me, there's a lot wrapped up in ‘influencer,’ and the word ‘influence’ doesn't suit me much. We're not influencing anything whatsoever; that's why I prefer talking about raising awareness. My content isn't meant to sell something—although that's not always the goal for influencers either.

(Chloe, Switzerland)

Instead, they emphasised their healthcare credentials (doctors, nurses, health coaches) and/or lived experience as the basis for their authority.

I am a medical doctor. I've got many years experience, published papers, published these books for people. So yeah, I've got those credentials behind me. I'm not someone that just thinks that they know stuff.

(Odette, UK)

In some cases, credibility was drawn from fields adjacent to healthcare, and their role within the community was closely tied to this identity.

Influencer seems a little bit like less professional. So, yeah, I like to think of myself as an educator, just because I put a lot of research, like I'm a scientist, and I put a lot of research into the things I share.

(Maggie, UK)

### 3.4 Theme 2: Mission as Motivation

Participants were intrinsically and extrinsically motivated to create impact and foster change in women's health, often highlighting gaps in healthcare delivery and access.

From the outset, it was always about helping people know things that might make them improve their relationship with their their body and themselves really, where you know, they could access it before things spiralled or, you know, accessing it, if things are spiralling but they don't have access to services because they're so limited.

(Joanna, UK)

Motivation stemmed from multiple sources, including personal experiences with health conditions and healthcare.

Knowing that there's so many people out there who are just starting this journey, or they're in the depths of it, like I was in 2018, and knowing that we can try to help cut down and shorten someone's path to finding relief or answers and support like that, to me, is what, what drives me every day.

(Theresa, Canada)

A sense of duty to represent the community with which they identified was cited as a primary motivation for launching a social media profile:

I, number one, didn't become a urologist myself until I saw a black woman who was a urologist, and I was really amazed. And I am not shy in front of the camera. And so I thought, you know, here I am. I've achieved—my goal is to, if you will, let me share this with the world. I knew the power of representation and just being visible to one woman, person of colour, other and inspiring them. I felt like it was my duty.

(Jade, United States)

Many participants described their content creation work as mission‐driven rather than commercially motivated. They felt a sense of responsibility for their followers and the desire to improve outcomes via knowledge transfer and empowerment.

Women's health is my passion. And I believe that we simply have to work much more individually here, separate from the perspective of men. We women tick very differently, we women have a completely different body. And the knowledge must be shared, absolutely.

(Ela, Germany)

So my mission with my work is to improve the global health of women by empowering women with knowledge so they can make the right treatments for them and their future health.

(Rebekah, UK)

This sense of responsibility extended to helping others take responsibility for themselves and their own health.

I want to help people take responsibility for their own health, because nobody's going to do it for you, not even I can do it for you. So I show you the guide posts. Give you the pathway, but you have to walk along it.

(Cate, Australia)

### 3.5 Theme 3: Leveraging Reach

Participants reported what they perceived as evidence of mobilising followers, from witnessing heightened awareness to observing concrete health‐related actions.

Yes, they change their behaviour. I see that the information is being spread—they take what I say into account; sometimes they adopt it, sometimes they share the information themselves, orally with their loved ones. So I see that even if it's not 100% of what I say, a large part is adopted.

(Mila, Tunisia)

They described initiating behaviour change in their followers, such as seeing their community seeking medical appointments, advocating for themselves in clinical encounters, changing their health behaviours, pursuing diagnoses, and participating in research studies based on recommendations.

I don't shy away from giving people the facts and the understanding, hopefully that they can therefore, you know, find that motivation to make behavioural changes, which are what I'm about.

(Odette, UK)

Participants received regular feedback from followers describing specific actions taken: “because I heard you talking about this, I went to the doctor,” (Esther, Kenya); “she got the biological tests she needed,” (Aurelie, France); “I've been doing this for 3 weeks now and it has already improved,” (Ela, Germany).

On some things—typically books or podcasts—I think there's a concrete impact, that they'll go read something they wouldn't necessarily have read or listen to something. I think so, yeah. At least I've already gotten feedback, mostly on the Discord—people who say ‘Hey! I read this thing,’ ‘me too, I just read it,’ ‘me too,’ ‘me too.’

(Louise, France)

Some participants could point to measurable outcomes including improved clinical metrics in followers, successful research recruitment for studies they promoted, or increased treatment‐seeking rates.

I also regularly get thank‐you messages, saying ‘Lena, thanks. Thanks to this advice I now have fewer UTIs,’ or ‘I didn't know there's a vaccination against UTIs, thank you very much for that.’ So you do notice lifestyle changes.

(Lena, Germany)

### 3.6 Theme 4: Care‐Centred Gatekeeping

Participants functioned as rigorous gatekeepers who carefully evaluate content, particularly research opportunities, before sharing with their communities.

As long as I say I'm protecting my audience, you know, because that could be hugely damaging. You know, if you can imagine, you've sent some woman to some seedy back street clinic that wasn't as it was meant to be, type thing? Yeah, I just would be protective of my followers and audience.

(June, UK)

This gatekeeping was motivated by a strong sense of responsibility to protect vulnerable followers from poor‐quality research, excessive burden, or potential exploitation.

I would say if, number one, if it is authentic. Number two, if it is safe for my patients, because at the end of the day, I am a nurse, I do no harm. And number three, if it definitely aligns with my patients' health and how they benefit from it.

(Alina, Kenya)

Participants evaluated research on multiple dimensions: scientific quality and methodological rigour, participant burden versus benefit, ethical approval and data protection, alignment with community needs and values, and researcher credibility.

First of all the conductor of the study — it has to be a serious entity, because otherwise I'm not going to share it. I mean, if it's an entity that is recognised at a scientific level — a university rather than other research institutions — that's definitely a criterion. If it's done— now, if it's an influencer, clearly I don't even get involved because there has to be credibility, and then at least some guarantee on the use of the data. The seriousness of whoever is promoting it.

(Regina, Italy)

I want to know about their study. I want to know about them also, like, how long have they been, you know, conducting research. What university are they affiliated with? What hospital are they affiliated with? What's the motivation of the research? Just to make sure that wherever I'm directing my audience, like I said, I don't take it lightly that I have their trust. But wherever I'm directing my audience, that it's to a good place that has their heart in it. And yeah, for the good of society, yeah, and not for just the good of like, sales of vaccines and stuff you know, like, it needs to make sense.

(Cassie, Canada)

Many described personally vetting studies by reading protocols, testing surveys themselves, or assessing whether participation requirements were reasonable given their followers' circumstances.

I look at how the questionnaire is formulated, so if in my opinion the questions are relevant for the topic. I really appreciate studies that are inclusive, also in the options provided for the question of gender, that there isn't only male or female. So yes I also consider this. Obviously it's not that I automatically reject non‐inclusive studies, but I do prefer when there is inclusivity.

(Beatrice, Italy)

### 3.7 Theme 5: Values‐Led Reciprocity

Participants held diverse, often contradictory views on compensation for research collaboration, revealing that reciprocity cannot be standardised. Some categorically refused payment to maintain authenticity and avoid commercialising their relationship with followers, feeling that compensation ‘would raise some flags for me,’ (Madison, Canada) or that they would ‘be less inclined to share a study that I was getting compensation for sharing,’ (Joanna, UK). Some expected fair compensation, viewing their work as skilled labour deserving recognition and remuneration. Others navigated case‐by‐case, with decisions influenced by study type, time burden, mission alignment, and personal financial circumstances: ‘It depends on how much is being shared. How much time it takes,’ (Ela, Germany)

[Compensation is] important if there's work involved. So if I'm creating content in a blog post, and I'm putting the time and the energy into creating that, then I do feel that compensation is deserved for any influencer. If it's something that you're like, ‘Oh, can you share this paragraph’ and it's a copy and paste ‐ then I can do that. But any sharing like it takes our energy. It takes our time. So compensation, of course, is something that is, I would think any influencer who is sharing this information would probably consider.

(Theresa, Canada)

For those refusing payment, compensation threatened the trust‐based relationship with followers; it risked appearing as ‘selling’ research to their community. For those expecting payment, compensation signalled respect for their expertise and labour, particularly given the substantial unpaid time many invested in content creation.

Importantly, reciprocity extended beyond monetary compensation to include: access to research results, co‐authorship or acknowledgement, partnership in study design, community benefit from the research, and respectful collaboration.

The second thing we appreciate — which is not always done — is getting a follow‐up afterwards. Because that allows us to enrich our community with information. And it's true that we don't necessarily know upfront, but when researchers tell us ‘I can send you the results or the publications,’ even just the little emails — I love the small email that says ‘It's been published.’

(Aurelie, France)

I always ask, if we participate in the study, I disseminate the questionnaire among my followers, among our members, that one, you give us the study results, you give them also to us. Two, you cite our organisation in the study and so as the main source, because we know we would be the main source, whatever source they use, but especially in the urogenital field we are the largest organisation in Italy, so we know we would be the main source of data. And so we also ask to be included in the study, also to give other women the chance to get to know us, to know we exist, so it's an exchange.

(Aurora, Italy)

Participants wanted researchers to understand that value could be created through genuine partnership.

Being kept informed of what's happening and feeling considered—not part of the team, but not just ‘oh, you have to share this.’ Being listened to is always validating and makes you want to share even more. I think the more motivated we are to share a study, the more people will want to respond.

(Justine, France)

### 3.8 Theme 6: Collaboration Beyond Logistics

Participants desired practical support from researchers including ready‐made graphics, comprehensive study information and clear protocols, but emphasised that effective collaboration required more than logistical assistance. They wanted to be valued for their expertise and consulted as partners, not simply provided with materials to distribute.

Not just a link to the survey, but certainly like articulate wording around what it is, so like that value of what you're actually studying. You know, if you had a really great landing page with the anonymity sort of clause, but, certainly like marketing wording. I would probably do stories myself, like it would be my face. I would ‐ but maybe the person, the researcher, like putting a face to a name, is always really awesome, or the university symbol and things like that, like whatever is related to it to make it really authentic, to make it, you know, it's an actual place. It's an actual person.

(Cate, Australia)

Participants described ideal collaboration as involving: early consultation on study design and recruitment materials, input on messaging that would resonate with their communities, co‐creation of content, and ongoing communication throughout recruitment.

I think asking for our opinion before sharing the study, for example. Yes, that could work. I'm not saying be there all the time ‐ but having a say before it's shared, giving feedback, and having it considered where possible. We can have ideas that make the medical team think, and that can help advance the cause.

(Justine, France)

Many expressed disinterest in transactional approaches, pointing to researchers sending generic emails with survey links and expecting promotion without context, relationship, consultation or authenticity. They emphasised that their community knowledge, communication expertise, and understanding of patient barriers were valuable contributions that could strengthen research design and help recruitment efforts reach the right people, faster and more effectively than generic research message amplification alone.

There needs to be sympathy from the start. I think it's not enough to just send an email, ‘we have a study, please promote it.’ I think it's quite important to first have personal contact.

(Lena, Austria)

### 3.9 Positioning Themes Within Theoretical Frameworks

Two important insights emerged via an examination of the six themes against established theories of influencer effectiveness. First, influential health content creators demonstrate the same capabilities documented across literature on commercial influencers [12, 13, 15, 16, 17]. Second, novel findings around health content creator views on research collaboration also align with established theories, further confirming their validity [21, 22, 26]. Table 2 presents these alignments with Source Credibility Theory; Similarity‐Attraction Theory; and Social Exchange Theory, showing which theoretical constructs were reflected in each theme. Source Credibility Theory was evident in four themes, particularly where participants emphasised their professional credentials, lived experience, and community trust as foundations for influence.

**Table 2:** Themes and theoretical frameworks.

Theme	Source credibility theory	Similarity‐attraction theory	Social exchange theory
1. Educators, not influencers	Expertise, trustworthiness (credentials and lived experience)	Homophily (through shared lived experience)	
2. Mission as motivation			Intrinsic rewards, sense of purpose (influencer‐follower)
3. Leveraging reach	Credibility enables action (‘purchase intention’ as action)	Similarity increases persuasiveness	Mutual benefit: followers receive guidance leading to improved outcomes; creators fulfil mission through impact
4. Care‐centred gatekeeping	Protecting trustworthiness	Protecting shared community values and beliefs	
5. Values‐led reciprocity	Authenticity maintained through shared values		Researcher‐influencer expectations for values‐aligned exchange
6. Collaboration beyond logistics		Researcher‐influencer value alignment increases willingness to collaborate	Researcher‐influencer exchange: expertise and access for genuine consultation and recognition

*Note:* The six themes mapped against established theories of influencer effectiveness, including: Source Credibility Theory, Similarity‐Attraction Theory and Social Exchange Theory.

Similarity‐Attraction Theory also appeared in four themes, with participants describing how shared experiences and values created connections with both followers and potential researcher partners. Social Exchange Theory operated across three themes but at different relational levels: influencer‐follower exchanges in Theme 2, researcher‐influencer compensation exchanges in Theme 5, and researcher‐influencer partnership exchanges in Theme 6. Notably, Theme 3 (Leveraging reach) represented the convergence of all three theoretical mechanisms, including credibility, similarity, and exchange relationships, working together to mobilise the follower action described by participants. Theme 6 (Collaboration beyond logistics) extended these theoretical frameworks from influencer‐follower relationships to illuminate researcher‐influencer partnership dynamics, demonstrating how Similarity‐Attraction and Social Exchange Theories also operate at this partnering level.

## 4 Discussion

### 4.1 Principal Findings

This study explored how women's health and science content creators make decisions about research recruitment collaboration with analysis revealing six key themes. Creators position themselves as educators with expertise grounded in professional credentials, lived experience, or both (1). Driven by their personal mission (2), they argue that they have the capacity to mobilise follower behaviour change (3). A strong sense of responsibility translates into careful gatekeeping and evaluation of research opportunities through multiple lenses including scientific quality, ethical standards, participant burden, and community benefit, to protect vulnerable followers from exploitative or low‐quality research (4). When creators do collaborate with researchers, their preferences range from quick correspondence and ready‐made materials to genuine partnerships that recognise their contributions beyond promotion. Reciprocal research relationships that balance intrinsic rewards, fair compensation, and values alignment were often sought (5), as were genuine partnerships that recognise their extensive expertise (6). Influential health content creators hold a unique position within the online community that enables them to address critical gaps in traditional research recruitment through three key mechanisms: established presence within and engagement with online patient communities; credibility earned through professional credentials and shared lived experience; and perceived capacity to mobilise followers from awareness to action, including research participation.

Specifically, these capabilities have the potential to overcome medical mistrust, restricted access to patient populations outside institutional healthcare settings, and challenges in converting research candidates to research participants [8, 33, 34]. Consistent with the theoretical mapping presented in Table 2 (Section 3.9), participants' credibility drew on two complementary sources: professional credentials, which granted scientific and clinical authority, and lived experience, which conferred authenticity and fostered homophily with followers under Similarity‐Attraction Theory [21, 22, 25]. Whether credibility stemmed from professional expertise, lived experience, or their combination, health content creators positioned themselves as trustworthy sources whose recommendations carried weight with their communities. Their identities were grounded in helping rather than selling, consistent with their rejection of the ‘influencer’ label in favour of ‘educator’ or ‘advocate.’ Participants described this combined credibility and perceived similarity as translating into a self‐reported capacity to mobilise follower action, reflected in follower‐reported behaviours including seeking medical appointments, advocating for themselves in clinical encounters, pursuing diagnoses, and participating in research studies. At the creator‐follower level, this mission‐driven social exchange [26] sustained content creation despite often substantial unpaid effort, with creators investing time, expertise, and emotional labour in return for intrinsic rewards including gratitude, a sense of impact, and validation of their experiences.

When researchers approach creators for recruitment collaboration, a further exchange relationship emerges. Decisions about monetary compensation vary widely, with some creators refusing payment in order to maintain authenticity with followers, others expecting fair recognition of their labour and access to their community, and many navigating case‐by‐case decisions based on study alignment and time burden. Beyond compensation, some creators emphasised expectations for genuine partnership characterised by early consultation, meaningful input into study design and recruitment approaches, and recognition of their community expertise. Collaboration was more likely when researchers demonstrated values alignment, respect for the community, and understanding of patient experiences, extending the influencer‐follower application of Similarity‐Attraction and Social Exchange Theory [22, 26], to illuminate partnership formation as a distinct process requiring mutual respect and shared values.

### 4.2 Practical Recommendations for Strategic Content Creator Partnerships

Influential health content creators possess community expertise, self‐reported capacity to influence health behaviours, and gatekeeping responsibility toward populations who may be vulnerable due to their experiences with chronic illness; capabilities that position them as potential partners rather than simply promotional channels. While they may be open to one‐off requests to share research recruitment materials, deeper collaborations allow researchers to benefit from content creator input across all stages of the research lifecycle. Optimisation of this input requires early engagement, relationship‐based outreach, and recognition of creators as expert partners with valuable contributions to study design and recruitment strategy. Budgetary considerations for these strategic partnerships should occur early in the research planning stages alongside the assessment of other recruitment channels and public and patient involvement and engagement (PPIE) [35]. Feasibility within typical academic budgets will vary, though findings from Theme 5 suggest many creators do not require financial compensation, allowing tailored partnerships to be built across a range of resource levels. The following recommendations are grounded in participant perspectives, combining creator‐requested practices with pragmatic propositions extending this input into actionable guidance; further empirical testing would help confirm their effectiveness in practice. Table 3 presents recommendations for researcher‐influencer collaboration across planning, recruitment, and post‐recruitment phases.

**Table 3:** Recommendations for researcher–creator partnerships.

Research phase	Recommendations
Planning	Identify relevant creators based on content focus, audience demographics, and values alignment Initiate relationship‐building without immediate requests, and determine to what extent creators may wish to be involved in the research process If appropriate, consult creators on study design, recruitment materials, and community barriers Discuss reciprocity preferences early (payment, co‐authorship, acknowledgement, results access) and add any remuneration to the research budget
Recruitment	Provide comprehensive study information (purpose, methods, eligibility, burden/benefit, ethical approval) to allow creators to evaluate the study Offer ready‐made materials while allowing adaptation to creator's style Assign a dedicated research contact and provide regular updates to the creator Discuss reciprocity preferences early (payment, co‐authorship, acknowledgement, results access)
Post‐recruitment	Share results in plain language formats to enable the creator to update their community about study progress or outcomes Offer co‐dissemination opportunities Acknowledge creator contributions publicly if requested Maintain contact beyond single studies if appropriate for future work
All research phases	Respect expertise (professional or experiential) by encouraging feedback Welcome gatekeeping as quality assurance Individualise approaches to each creator Build genuine relationships, not transactional exchanges Maintain transparency about goals and expectations

*Note:* Recommendations for researcher‐influencer collaboration are provided and split into three research phases: planning, recruitment, and post‐recruitment. Additional recommendations for all recruitment phases are included.

### 4.3 Ethical Considerations

Although creator‐researcher partnerships provide opportunities, they also carry risks that warrant careful consideration. Table 4 summarises key ethical considerations specific to this recruitment approach, alongside safeguards to mitigate each.

**Table 4:** Ethical challenges and safeguards for researcher–creator partnerships.

Ethical consideration	Arising from	Safeguard
Undue influence and compromised autonomy [36, 37, 38, 39]	Parasocial trust may blur the line between community belonging and voluntary participation, particularly for those who have experienced healthcare dismissal and feel validated by community voices	Clear separation between creator‐led recruitment exposure and the consent process; independent researcher contact pathways distinct from influencer channels
Therapeutic misconception [40, 41]	Trusted creators promoting research may lead followers to conflate participation with treatment, overestimating benefits or underestimating risk	Consent processes that explicitly distinguish research from clinical care; disclosure of any creator‐researcher relationship or compensation
Selection bias and equity [42, 43, 44, 45, 46]	Reliance on digitally engaged, research‐favourable creators may exclude less visible communities or those with lower digital access or comfort	Hybrid recruitment combining creator‐led and traditional methods; transparent reporting of sample limitations
Inequitable researcher‐creator partnership [47, 48, 49]	Transactional models that treat creators solely as promotional channels fail to respect their community expertise and advocacy priorities	Reciprocal disclosure of roles; meaningful consultation on research communication; recognition of creator expertise in formal research outputs

*Note:* Key ethical risks associated with recruiting research participants via health content creators, alongside safeguards to mitigate each.

### 4.4 Strengths and Limitations

This study offers the first comprehensive exploration of health content creator decision‐making for research recruitment collaboration. Participants' willingness to take part in this study indicates an existing interest in research engagement, meaning the sample is inherently biased toward creators favourably disposed to research. However, this is precisely the population researchers need to identify when seeking creators as future recruitment partners, making this bias a feature of the sample's relevance rather than a limitation to be corrected. The selection pattern is also important to consider as creators favourably disposed to research may be more likely to articulate a coherent educator identity (Theme 1) and openness to research partnership (Theme 6); creators who are sceptical of academic research, or whose activity is more commercially oriented, for example, reliant on brand partnerships or sponsored content, may have been less inclined to volunteer, and are therefore likely underrepresented in this sample. As a result, the extent to which the ‘educator, not influencer’ identity and the openness to collaboration described here generalise to health content creators with more commercial or research‐sceptical orientations remains a core topic for exploration. The strong alignment between findings and established theoretical frameworks strengthens confidence in the results, demonstrating that health content creator influence operates through recognised psychological mechanisms while extending their application to novel contexts including researcher‐influencer partnerships.

While participants were predominantly from the Northern hemisphere, the multilingual nature of the sample and the variance in follower sizes enhance transferability of findings and demonstrated consistent patterns across diverse contexts. However, the study was not designed to capture cultural or national variation, and differences in institutional trust, health information‐seeking behaviour, or content creator norms across regions may have shaped participants' experiences in ways not identifiable through this analysis; exploring this systematically would be a valuable direction for future research. The study focused solely on women's health and science, yet this specificity enabled deep examination of online health content creator perceptions in niches that are frequently dismissed or under‐researched. Moreover, inclusion of both healthcare professionals and patient advocates within this focused domain revealed convergent themes across different expertise types, suggesting these perspectives are widely held rather than discipline‐specific. Further comparative research could build on these findings and examine whether partnership dynamics, educator self‐positioning, gatekeeping practices, and recruitment effectiveness differ across varying levels of content specificity, professional credentials, and across conditions with different levels of online community engagement.

Evidence of behaviour change capacity was self‐reported rather than objectively measured through platform analytics. However, this approach is appropriate for exploratory qualitative research aimed at understanding perspectives and decision‐making processes. The consistency of reports across participants using different languages and platforms, and with varied follower sizes supports the robustness of these findings. Further work is needed to explore and measure the extent to which health content creators can drive research‐specific behaviour change among their followers. Future research directions include longitudinal studies tracking partnership evolution and sustainability, and mixed‐methods designs combining creator interviews with follower surveys and recruitment metrics to quantify effectiveness. In addition, examination of researcher perspectives to illuminate both sides of collaboration dynamics, and economic analyses comparing cost‐effectiveness across recruitment channels will furnish valuable insights.

## 5 Conclusion

Research recruitment faces a critical bottleneck: traditional methods cannot access the millions of patients who congregate in online health communities, where medical mistrust and dismissed conditions drive people to seek peer‐validated information outside formal healthcare channels. Through multilingual qualitative interviews with 22 health content creators with follower numbers between 1300 and 750,000, this study reveals that these creators possess three capabilities traditional recruitment channels fundamentally lack: established presence within online patient communities where target populations have an active interest in a specific health topic, credibility derived from professional expertise, shared lived experience, or both, and a self‐reported capacity to mobilise followers from awareness to concrete health actions, including research participation. Effective influence mechanisms vary by credibility source: for creators with lived experience, influence operates through relatability and recognition, while professionally credentialed creators provide trusted clinical authority.

For researchers facing enrolment challenges, particularly in women's health and patient populations excluded from traditional recruitment pathways, these findings provide a critical evidence base justifying the importance of transforming ineffective social media advertising practices into strategic partnerships. Recognising health content creators as expert partners with community knowledge, gatekeeping responsibility, and mobilisation capacity reconceptualises their value in connecting research with the populations it aims to serve. This study demonstrated that meaningful access to hard‐to‐reach patient populations is achievable through ethical, relationship‐based partnerships with health content creators, offering a scalable, community‐grounded alternative to traditional recruitment methods that are poorly positioned to engage these communities.

## Author Contributions

**Melissa L. Kramer:** conceptualisation, investigation; writing – original draft, methodology, formal analysis, project administration, data curation, resources. **Bianca Serio:** writing – review and editing, project administration, data curation. **Aileen K. Ho:** conceptualisation, supervision, writing – review and editing. **Katherine A. Finlay:** conceptualisation, writing – review and editing, supervision, data curation, formal analysis, methodology.

## Funding

The authors have nothing to report.

## Ethics Statement

Ethical approval was received from the University of Reading (approval number: 2025‐023‐KF).

## Conflicts of Interest

Melissa L. Kramer reports a relationship with Live UTI Free Limited that includes equity or stocks. Bianca Serio reports a relationship with Live UTI Free Limited that includes consulting or advisory services. No funding was received for this research, and Live UTI Free Limited does not have a financial relationship with any of the participants in this research. If there are other authors, they declare that they have no known competing financial interests or personal relationships that could have appeared to influence the work reported in this paper.

## Supporting information


Supporting File S1

Supporting File S2

## Data Availability

The data that support the findings of this study are available on request from the corresponding author. The data are not publicly available due to privacy or ethical restrictions.
