# Supplementary material for: Educators, Not Influencers: Health Content Creator Perspectives on Research Recruitment Collaboration
Source: Health Expect. 2026 Aug 20;29(4):e70836. doi: 10.1111/hex.70836 (PMC13491538; doi:10.1111/hex.70836)
Supplement: Supplementary file 1 [file HEX-29-e70836-s001.pdf]

# S1. English Interview Topic Guide

## Understanding health content creator motivations and decision-making processes.

1. Can you tell me about your background and what motivated you to start sharing women's health or science content?
    a. What do you aim to achieve by sharing health or science-related content?
    b. What keeps you motivated to continue this content-sharing work?

2. What term would you use to describe yourself or your role in the online community?
    a. How do you feel about the term ‘influencer’?

3. Based on your own observations, how would you describe the typical interactions or sentiments of your audience around content about scientific research studies?
    a. What do you think personally motivates your audience to interact with research-focused content?

4. In what ways does your audience engage with you and your content, for instance, likes, comments, shares, link clicks, saves, or no engagement at all?
    a. Can you share your observations regarding how your content has the ability to impact the behaviour of your audience?

5. Can you tell us about where your audience shares your content and what you’ve observed?
    a. How would you describe the types of audience members who are most likely to share your content?

6. How do you decide what content to post and, therefore, what not to post?
    a. What do you feel are the limitations of sharing health and science-related content on social media?

7. To what extent have you shared opportunities for your audience to participate in research?
    a. Can you tell me more about why you have/haven't done so?
    b. In what ways do you think your influence may prompt people to consider participating in research?

8. What criteria would/do you consider before deciding whether you feel comfortable posting a research participation opportunity?
    a. What are your concerns about sharing this type of content, if any?
    b. What, for you, are the ethical considerations involved in deciding whether to post particular content?
    c. How important is the level of compensation in your decision-making process around sharing research participation content?

9. How would you feel about researchers and recruiters reaching out to you about working with you on sharing research participation opportunities?
    a. What support would you require from the researcher in order to share research participation content?
