# Supplementary material for: Educators, Not Influencers: Health Content Creator Perspectives on Research Recruitment Collaboration
Source: Health Expect. 2026 Aug 20;29(4):e70836. doi: 10.1111/hex.70836 (PMC13491538; doi:10.1111/hex.70836)

S2. Recruitment Procedure Flow Diagram

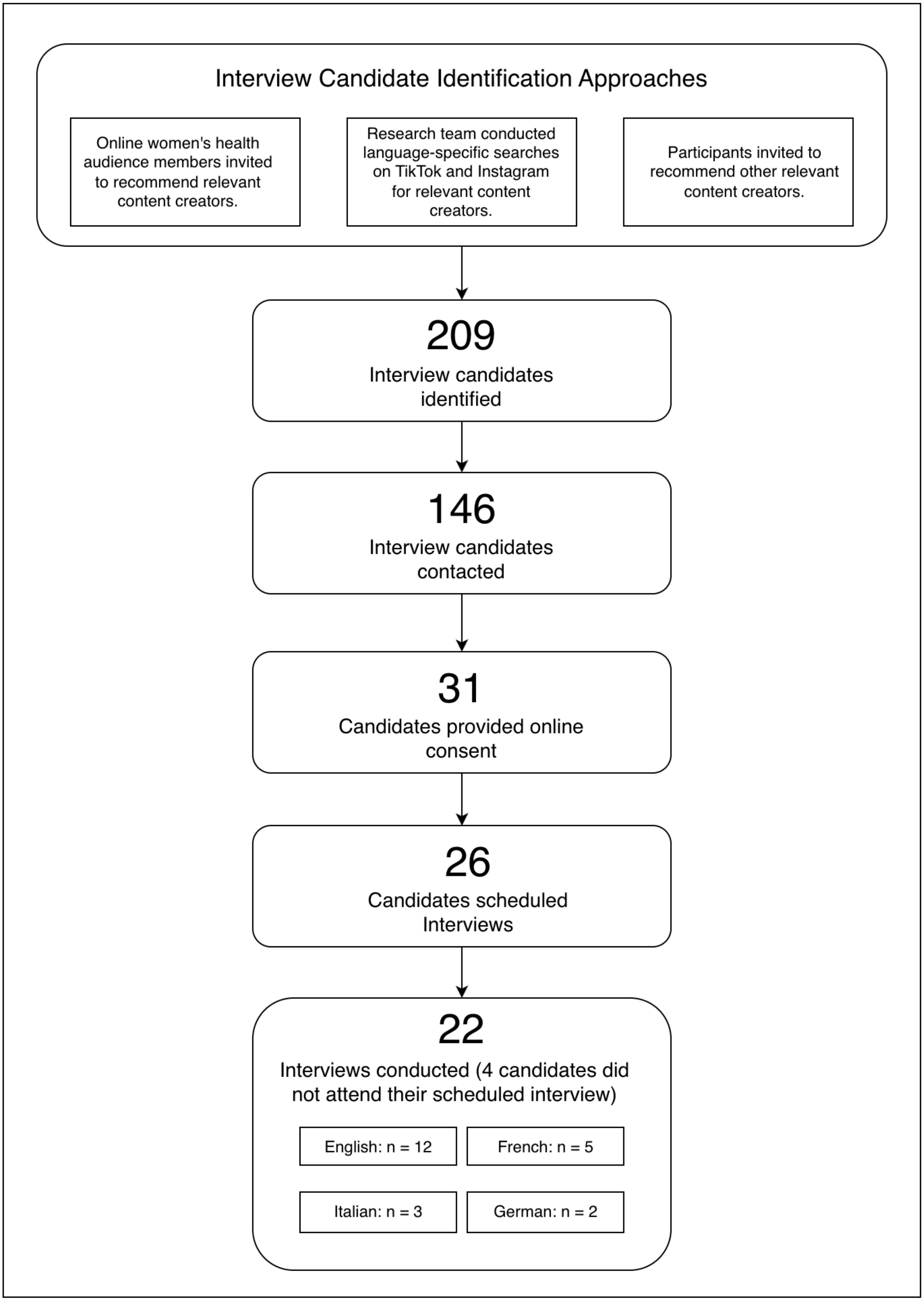

Supplement: Supplementary file 2 [file HEX-29-e70836-s002.pdf]
